# Continuous Positive Airway Pressure plus Mandibular Advancement Therapy (PAPMAT): study protocol for an adaptive randomised crossover trial comparing the benefits and costs of combining two established treatments for obstructive sleep apnoea

**DOI:** 10.1186/s13063-023-07484-w

**Published:** 2023-07-24

**Authors:** Martin Law, Sofia Villar, Nicholas Oscroft, Victoria Stoneman, Julia Fox-Rushby, Yi-Da Chiu, Jo Steele, Thomas Devine, Claire Francis, Thomas Claydon, Graham Hill, Ka Keat Lim, Joanna Rayner, Mandy Williams, Emma Spires, Timothy Quinnell

**Affiliations:** 1 MRC Biostatistics Unit, Cambridge University, Cambridge, UK; 2 Respiratory Support and Sleep Centre, Royal Papworth Hospital, Cambridge, UK; 3 Papworth Trials Unit Collaboration, Royal Papworth Hospital, Cambridge, UK; 4 Department of Population Health Sciences, King’s College London, London, UK; 5 Independent Consultant, Cambridge, UK; 6 Independent Consultant, Southend-on-Sea, UK

**Keywords:** Sleep apnoea, CPAP, MAD, Combination, Crossover, Sample size re-estimation, Cost-effectiveness

## Abstract

**Background:**

Obstructive sleep apnoea (OSA) involves repeated breathing pauses during sleep due to upper airway obstruction. It causes excessive daytime sleepiness and has other health impacts. Continuous positive airway pressure (CPAP) therapy is effective first line treatment for moderate to severe OSA. Unfortunately, many patients have difficulty tolerating CPAP and pressure intolerance is probably an important contributing factor. Mandibular advancement devices (MAD) are an alternative to CPAP. They are worn in the mouth during sleep to reduce airway obstruction. There is some evidence that, when used in combination with CPAP, MADs improve airway anatomy enough to reduce the CPAP pressure required to treat OSA and that this combination therapy could improve CPAP adherence.

**Methods:**

Consecutive patients starting on CPAP for moderate to severe OSA will be recruited at a regional NHS sleep service. Patients with high CPAP pressure requirements after initial titration, who satisfy all entry criteria and consent to participate, will undertake a 2-arm randomised crossover trial. The arms will be (i) standalone CPAP and (ii) CPAP + MAD therapy. Each arm will last 12 weeks, including 2 weeks acclimatisation. CPAP machines will be auto-titrating and with facility for data download, so the impact of MAD on CPAP pressure requirements and CPAP adherence can be easily measured. The primary outcome will be CPAP adherence. Secondary outcomes will include measures of OSA severity, patient-reported outcome measures including subjective daytime sleepiness, quality of life, and treatment preference at the trial exit and health service use. Cost-effectiveness analyses will be undertaken.

**Discussion:**

If the intervention is shown to be effective and cost-effective in improving adherence in this standard CPAP-eligible OSA patient population it would be relatively straightforward to introduce into existing OSA treatment pathways, within the wider NHS and more widely. Both MAD and CPAP are already used by sleep services so their combination would require only minor adjustments to existing clinical pathways. It would be straightforward to disseminate the results of the study through regional, national, and international respiratory meetings. The health economics analysis would provide cost-effectiveness data to inform service planning and clinical guidelines through policy briefing papers, including those by NICE and SIGN.

**Trial registration:**

PAPMAT was registered with ISRCTN prior to recruitment beginning (ISRCTN Registry 2021): https://www.isrctn.com/ISRCTN33966032. Registered on 17th November 2021.

## Administrative information

Note: the numbers in curly brackets in this protocol refer to SPIRIT checklist item numbers. The order of the items has been modified to group similar items (see http://www.equator-network.org/reporting-guidelines/spirit-2013-statement-defining-standard-protocol-items-for-clinical-trials/).

**Table:** 

Title {1}	Continuous Positive Airway Pressure plus Mandibular Advancement Therapy (PAPMAT): study protocol for an adaptive randomised trial comparing the benefits and costs of combining two established treatments for obstructive sleep apnoea
Trial registration {2a and 2b}.	**Study IRAS ID:** 296163 **Funder Identification:** NIHR201124 ISRCTN: 33966032 [1]
Protocol version {3}	Date: 5^th^ April 2023. Version 5.0
Funding {4}	**Funder:** National Institute of Health Research: NIHR Research for Patient Benefit (NIHR201124)
Author details {5a}	Affiliations of protocol contributors: M.L.^1,2^, T.Q.^1^, N.O.^1^, S.V. ^1,2^, V.S.^1^, J.F.R.^3^, Y.D.C.^1,2^, J.S.^1^, T.D.^1^, C.F.^1^, S.T.C.^4^, G.H., K-K.L.^3^, J.R.^1^, M.W.^1^, E.S.^1^ 1 Royal Papworth Hospital NHS Foundation Trust. 2 MRC Biostatistics Unit, University of Cambridge 3 Department of Population Health Sciences, King’s College London 4 NHS England Contributions of protocol contributors: TQ acquired funding with NO, SV, VS, JFR, YC, JS, CF, SC & GH. TQ wrote the initial protocol draft and oversaw the background scientific review, which was undertaken and drafted by CF. ML formatted the protocol manuscript for publication with respect to SPIRIT checklist. SV, YDC and ML provided statistical expertise in clinical trial design. JFR and KKL developed and drafted the health economic contributions. All authors contributed to refining the study protocol and approved the final manuscript
Name and contact information for the trial sponsor {5b}	Royal Papworth Hospital NHS Foundation Trust. R&D ID P02738 Dr Timothy Quinnell Respiratory Support and Sleep Centre, Royal Papworth Hospital NHS Foundation Trust, Cambridge, CB2 0AY, UK. tim.quinnell@nhs.net
Role of sponsor {5c}	The sponsor is responsible for study design; collection, management, analysis and interpretation of data; writing of the report; and the decision to submit the report for publication. The sponsor will have ultimate authority over these activities. The funder had no role in the design of this study and will not have any role during its execution, analyses, interpretation of the data, or decision to submit results.

## Introduction

### Background and rationale {6a}

This study will assess an intervention that could enhance the effectiveness of an existing NICE-recommended therapy for obstructive sleep apnoea (OSA). Both mandibular advancement devices (MAD) and continuous positive airway pressure (CPAP) therapy are part of NHS clinical services. Should the results prove effective and cost-effective the new ‘combination therapy’ could be rapidly introduced as an additional, efficient tool to help the significant number of patients who struggle with ‘monotherapy’.

OSA is caused by closure of the upper airway during sleep due to excessive muscle relaxation. Pauses in breathing caused by total (apnoeas) or partial (hypopnoeas) airway closure cause oxygen levels to drop and brief awakenings that result in excessive daytime sleepiness. OSA impacts all aspects of life, including social and work performance and driving safety, and impairs quality of life (QoL) [2]. OSA has other important health effects. It is linked to high blood pressure [3] and a 2.5 times higher risk of developing cardiovascular disease including heart attack and stroke [4]. OSA is associated with abnormal heart rhythms including atrial fibrillation, which itself undermines QoL and daytime functioning, and increases risks of stroke and road traffic accidents.

OSA is straightforward to diagnose and is treated with CPAP. A patient wears a mask connected to a small electric air pump that generates pressure to keep the throat open during sleep. CPAP is highly effective in treating OSA, improving daytime sleepiness, functional status and QoL. It is a treatment of choice for moderate-to-severe OSA [5, 6].

Unfortunately, not everyone tolerates CPAP and adherence rates range from 20 to 83%, so untreated OSA is prevalent [7]. Uncomfortably high pressure is a possible reason for CPAP intolerance. Patients start on a low pressure which is increased until OSA is controlled. For some patients the required pressure is higher than they can tolerate, making CPAP difficult to sleep with and defeating the purpose of treatment. In a nationwide survey of CPAP users, we found 68% had had difficulty using CPAP due to the pressure being delivered (details below). One method to try to improve CPAP tolerance is to use an auto-titrating CPAP machine. These aim to make CPAP more tolerable by continually monitoring for apnoeas and adjusting pressure according to whether they are occurring. Meta-analyses suggest auto-CPAP leads to a small improvement in CPAP adherence, although average pressure delivery is not much different to fixed pressure machines. Whilst auto-titration may help some patients to tolerate CPAP, it does not address all pressure-related issues and in some patients the pressure needed to control OSA exceeds CPAP's capacity, leading to ‘breakthrough’ OSA. More expensive machines deliver much higher pressures, but these are not available in every NHS service and there is no robust evidence that they are better tolerated [7].

MAD are worn in the mouth during sleep. They treat OSA by advancing the lower jaw and increasing airway space. MAD are not as good as CPAP at treating OSA. However, they are useful in milder diseases, are cost-effective [8] and sometimes help people with more severe OSA who cannot tolerate CPAP [5, 8].

Combining MAD with CPAP could potentially open the airway enough to allow CPAP pressure to be reduced. This could allow patients requiring higher CPAP pressures to tolerate treatment better and use CPAP more, increasing the chance of improving OSA symptoms and avoiding longer-term health impacts. If this study shows combination CPAP-MAD does reduce CPAP pressures, then it would also establish the utility of this intervention for improving the experiences of patients with ‘breakthrough’ OSA.

Up to 24% of middle-aged men and 9% of women have at least mild OSA (apnoea–hypopnoea index [AHI]) ≥ 5/h) (Young et al. 1993). Two per cent to 7% of adults have associated excessive daytime sleepiness, known as OSA syndrome (OSAS). Untreated OSA has a substantial economic burden in terms of reduced work productivity, increased road traffic accidents [9] and health care costs, especially for cardiovascular disease and stroke [10]. For example, older and middle-aged patients with OSA were shown to have roughly double the healthcare costs of aged-matched controls [11] which were reduced considerably with effective treatment [12]. However, the limited utility of other treatments for more severe OSA means that patients who cannot tolerate CPAP often remain untreated, so new ways to modify CPAP pressure delivery are still needed [13].

If the results of this study show CPAP-MAD combination therapy to be both effective and cost-effective it would be straightforward to make this treatment widely available. Results of our national patient survey suggest there are a significant proportion of OSA patients around the UK who need help and could benefit. Successfully treating these patients would not only improve their symptoms but could also reduce risk of longer-term conditions associated with untreated OSA, such as stroke. At population level, there is strong evidence that CPAP adherence is cost-effective [14] and superior to no treatment after a minimum of 2 years’ treatment [15]. It has been estimated that effectively treating all OSA could save the NHS roughly £1000 per patient [16]. The British Lung Foundation commissioned a report in 2014 which estimated that a total of £55 million and 40,000 QALYs annually could be saved if all people with moderate to severe OSA were diagnosed and treated with CPAP [17].

### Objectives {7}

**Table:** 

Objectives		Outcome measures
**Primary objective**	Does combining MAD with CPAP therapy make it easier for patients to tolerate CPAP, thereby increasing adherence to treatment?	Difference in CPAP adherence (hours per night) between treatment arms. Relevance: adherence to CPAP should aid OSA
**Secondary objectives**	Does combining MAD with CPAP therapy reduce CPAP pressure requirements?	• Difference in mean CPAP pressure between treatment arms. Relevance: high CPAP pressure is a possible reason for CPAP intolerance
	Does combination therapy objectively improve whole-night OSA control or office Blood Pressure compared to CPAP alone?	• Between-arm 4%ODI and AHI differences^a^ • Diastolic and systolic blood pressure
	Bespoke measurement of patient-specific resource and health service use	• Length of telephone support between arms • Time preparing and supporting/refitting MAD/CPAP • Use of medication and diagnostic tests • Visits to secondary care and use of primary and community care
	Does combination therapy improve patient-reported outcome measures (PROMs) compared to CPAP alone?	Patient-reported outcome measures: • Epworth Sleepiness Scale score (ESS) • Quality of life measured with Functional Outcomes of Sleep Questionnaire (FOSQ)/EuroQoL (EQ-5D-5L)/Short Form-36 (SF-36) • Pittsburgh Sleep Quality Index (PSQI) • Patient satisfaction and treatment preference • Side effects
	Is combination MAD with CPAP therapy cost-effective compared with CPAP alone?	• EQ-5D-5L and SF-6D quality-adjusted life years • Health service use

^a^From WatchPAT home sleep study worn for one night within the final week of each treatment arm

### Outcomes in detail

**Table:** 

Domain	Specific measurement	Specific metric	Aggregation method	Time point
CPAP adherence	Time spent each night using CPAP	Value each night	Continuous: each measurement used in mixed model	Downloaded at Baseline, start of each treatment and at each night (after 2 week’s acclimatisation per treatment)
CPAP pressure	Mean pressure measured each night on CPAP machine	Value each night	Continuous: each measurement used in mixed model	Downloaded at Baseline, start of each treatment and at each night (after 2 week’s acclimatisation per treatment)
4% ODI	Frequency of drops in oxygen saturation by at least 4% from baseline per hour of sleep, measured using WatchPAT home sleep study	Value per hour of a single night	Mean value	One night in final week, per treatment
WatchPAT—AHI	Frequency of apnoeas and hypopnoeas per hour of sleep, measured using WatchPAT home sleep study	Events per hour at a time point of a single night	Mean value	One night in the final week, per treatment
CPAP—AHI	Frequency of apnoeas and hypopnoeas per hour of sleep, acquired from download from CPAP machine	Events per hour	Mean and median value	Collected with CPAP download at baseline, at start of treatment (visit 3), then reported for both final 4 weeks of treatment and full 10 weeks of treatment
Blood pressure	Systolic blood pressure	Value at a time point	Continuous: paired difference	Visit 3 and end of each treatment period
	Diastolic blood pressure	Value at a time point	Continuous: paired difference	Visit 3 and end of each treatment period
Length of telephone support	Frequency of telephone support	Total per treatment	Continuous: paired difference	End of each treatment period
Use of anti-hypertensive medication	Type, dose, duration of medications used	Cost per treatment for each medication type	Continuous. Multiplied by unit cost and aggregated to total cost per patient	Visit 3 and end of each treatment period
Use of diagnostic tests – Watch PAT	Number of each type of diagnostic test used	Cost per treatment	Continuous Multiplied by unit costs and aggregated to total cost per patient	End of each treatment period
Sleepiness	ESS score	Value at a time point	Continuous: paired difference	Visit 3 and end of each treatment period
Quality of life	FOSQ	Value at a time point	Continuous: score	Visit 3 and end of each treatment period
	EuroQoL-VAS	Value at a time point	Continuous: score	Visit 3 and end of each treatment period
	EQ-5D-5L	Value at a time point	Continuous: score	Visit 3 and end of each treatment period
	SF-36 scores and SF6D values	Value at a time point	Continuous: score	Visit3 and end of each treatment period
Sleep quality	PSQI	Value at a time point	Continuous: score	Visit 3 and end of each treatment period
Treatment preference	MAD + CPAP or CPAP	Value at a time point	Binary	End of second treatment period
Ongoing treatment decision	MAD + CPAP or CPAP or other	Value at a time point	Trinary	End of second treatment period
Participant-reported side effects during the treatment period with MAD + CPAP	Individual participant reports side-effects experienced	Side-effects experienced Y/N then free text to add detail	Categorical: qualitative	End of second treatment period
Quality-adjusted life years	EQ-5D quality-adjusted life years	Utility value at a time point based on UK tariff for EQ-5D	Continuous. Area under the curve calculated as quality-adjusted life years. Mean difference	Visit 3 and end of each treatment period
Quality-adjusted life years	SF-6D quality-adjusted life years (subset of SF-36)	Utility value at a time point based on UK tariff for SF-6D	Continuous. Area under the curve calculated as quality-adjusted life years. Mean difference	Visit 3 and end of each treatment period
Health service use	Use of GP or nurse in-person, at home, via telephone or online, dentist, NHS111, trial helpline, ambulance, A&E, hospital outpatient, hospital overnight admission	Average (SD) use per service type per patient	Each service type valued using specific unit cost, with total cost for all services aggregated at patient level summarised as mean and SD at group level	Visit 3 (regarding last 1–12 months’ use and travel costs) and end of each treatment period

### Trial design {8}

This is a single-centre superiority trial comparing CPAP to CPAP + MAD (combination therapy), using an adaptive 2 × 2 randomised crossover design. The primary outcome is CPAP adherence. The treatment periods are 12 weeks each, with the first 2 weeks treated as an acclimatisation period.

## Methods: participants, interventions and outcomes

### Study setting {9}

Eligible patients will be adults referred to the Respiratory Support and Sleep Centre (RSSC) at Royal Papworth Hospital Foundation NHS Trust, UK, for CPAP titration.

### Eligibility criteria {10}

#### Inclusion criteria

- Adults with moderate to severe OSAS defined by a 4% oxygen desaturation index (4%ODI) or apnoea hypopnoea index (AHI) ≥ 15/h.
- Symptomatic daytime sleepiness (ESS) score ≥ 9
- Auto-titrated CPAP pressure ≥ 12 cm water

#### Exclusion criteria

- Inadequate dentition or other contraindication to MAD determined by clinician or trained CPAP provider.
- Co-morbid sleep disorder that might affect the patient’s ability to comply with treatment or benefit from therapy, or confound the interpretation of results.
- Unstable cardio-respiratory disease or other disorder/factor judged by the clinician to preclude trial participation due to safety concerns or significant potential to confound interpretation of results.
- Previous MAD or CPAP use (predating current treatment).
- Other reason for inability to comply with trial protocol.

Consecutive patients with moderate to severe OSA who are referred for CPAP titration and satisfy the initial eligibility criteria will be approached about the study at the time of their CPAP titration visit. All patients would qualify for CPAP therapy according to NICE guidelines and standard RSSC practice.

### Who will take informed consent? {26a}

Following initial screening for eligibility, potential participants are approached to introduce them to the trial by either a member of the research team or the CPAP Practitioners/Advanced Nurse Practitioners at the time of a patient’s CPAP initiation visit. The trial is explained, and a patient information sheet provided, and verbal agreement is gained to contact the patient to obtain their CPAP pressure reading once they have completed the initial CPAP titration. If the patient is still eligible and remains willing to take part in the trial, consent is taken by the Chief Investigator when the participant attends their six-week CPAP review.

Initial screening removes patients according to AHI/DI; ESS; quality of/out of date diagnostic study; presence of mixed/central sleep apnoea; previous use of CPAP/NIV; previous MAD use and other medical/sleep history related criteria. Once the pressure reading is confirmed to be ≥ 12 cm water the final eligibility criteria are checked by the Chief Investigator prior to consenting, including a dental and temporomandibular joint check for suitability to use a MAD.

### Additional consent provisions for collection and use of participant data and biological specimens {26b}

There are no plans to use participant data in ancillary studies. If this is subsequently considered then the data would be anonymised. Further consent would be sought otherwise. No biological specimens will be taken.

## Interventions

### Explanation for the choice of comparators {6b}

CPAP and MAD are both existing NICE recommended therapies for OSA on their own as “monotherapy”. Providing CPAP and MAD as a combination therapy could improve CPAP adherence and so help treat OSA.

### Intervention description {11a}

Participants with high CPAP pressure requirements after initial CPAP titration will undertake the 2–arm cross-over trial. They will be randomised to either standalone CPAP or CPAP + MAD therapy and will then cross over to the alternative arm. Each arm will last 12 weeks (including 2 weeks acclimatisation period). Auto-titrating CPAP machines that automatically adjust the pressure according to individual requirements will be used in the trial. This will enable evaluation of the impact of MAD on CPAP pressure requirements and telemonitoring functionality will allow remote data downloading.

### Criteria for discontinuing or modifying allocated interventions {11b}

Each participant has the right to withdraw from the study at any time. In addition, the Investigator may discontinue a participant from the study at any time if the Investigator considers it necessary for any reason including:

- Ineligibility (either arising during the study or retrospectively having been overlooked at screening)
- Significant protocol deviation (to be described in detail in the SAP)
- Significant non-compliance with study requirements
- Withdrawal of consent

The reason for withdrawal will be recorded in the CRF.

### Strategies to improve adherence to interventions {11c}

Adherence is recorded automatically and is the primary outcome of the study.

Participants will receive a telephone call if they have not sent their dental impression to the device manufacturer within 2 weeks. Our experience with a previous MAD study identified this as an area where additional support reduces the chance of a participant dropping out at an early stage if they have difficulty with moulding a dental impression. There is also telephone support available to participants mid-treatment phase to “touch base” and deal with any concerns.

### Relevant concomitant care permitted or prohibited during the trial {11d}

Any other medical care will be permitted as required. There are no prohibitions.

### Provisions for post-trial care {30}

Participants will be reviewed at trial end and offered to continue with either treatment arm. Alternative options for OSA treatment will be discussed as required and appropriate. Participants will return to long term follow-up under the clinical service. Participants are not expected to come to significant harm from involvement in the trial. However, if they do then the NHS Indemnity scheme would apply.

### Outcomes {12}

See the “Objectives {7}” section above.

### Participant timeline {13}

**Table:** 

	**Visit 1**	**Visit 2**	**Randomisation**	**Visit 3/*research visit 1/start of 1***^***st***^*** treatment***		**Visit 4/*research visit 2/end of 1***^***st***^*** treatment period***		**Visit 5/*research visit 3/end of 2***^***nd***^*** treatment period***	
				**CPAP 1st**	**CPAP-MAD 1st**	**CPAP 1st**	**CPAP-MAD 1st**	**CPAP 1st**	**CPAP-MAD 1st**
**Time interval of Visit**	Week 1	Weeks 6–7	Weeks 8–10	Weeks 9–10	Weeks 9–10	Weeks 19–20	Weeks 21–22	Weeks 31–32	Weeks 31–32
**Introduction to study and PIS**	X								
**Consent**		X							
**Medical History**		X							
**Physical exam/dental check**		X							
**Clinical review for CPAP pressure**		X							
**CRF completion**		X				X	X	X	X
**Study Questionnaires**					X	X	X	X	X
**MAD moulding**		X							
**MAD quality check**			X						
**Randomisation**				X	X				
**MAD/CPAP fitting**				X	X				
**Switch auto-CPAP**				X	X				
**Begin study intervention CPAP or CPAP + MAD**				X	X	X	X		
**CPAP data download**						X	X	X	X
**Overnight sleep study**						X	X	X	X
**Return of sleep diary**							X	X	
**Clinical review and treatment preference**								X	X
**Completion of service use questionnaires**								X	X

### Sample size {14}

A sample size of 64 patients was selected based on power (90%) and type I error (5%) considerations for the primary endpoint of average hours of CPAP usage within the treatment window of 10 weeks.

Using data from the TOMADO study [5], we estimated a pooled variance of 5.51 within pairs of observations. Using this estimate as a reference (assuming that patient variability between CPAP and CPAP-MAD will be similar) we obtain a sample size of 58 patients to detect an hour increment in the average use of the treatment (combination over standard of care). The sample size formula used is Eq. (9) of Siyasinghe and Sooriyarachchi [18]. To allow for estimated loss to follow-up (informed from TOMADO experience) we intend to randomise an additional 10% of patients to give a final sample size of 64.

### Recruitment {15}

The recruitment rate will be closely monitored by the project team and the Trial Steering Committee. The aim is to have 50% recruited by months 14–16 from when the trial opens for recruitment and an assessment of feasibility for recruitment will be undertaken at this time if recruitment is below 35%.

One potential area for modification if recruitment is low is the eligibility threshold for CPAP pressure. The chosen inclusion criterion of ≥ 14 cm water was informed by the limited published data and a survey of our CPAP patients’ pressure ranges. Pressure readings for all patients screened for the trial will be recorded and if necessary we will examine the recruitment uplift potential of reducing the threshold to, e.g. 13 or 12 cm water.

From the initial review of screening results during the project team meeting of September 28th 2022, it was evident there was a significant chance of missing potentially suitable participants through pressure threshold requirements. Of the 22 not eligible due to pressure, 18 had reading < 10 cm, one had reading 10–11 cm; two had reading 12–13 cm and one had reading 13–14 cm. Following communication with TSC, it was agreed to lower the eligibility threshold to ≥ 12 cm water and amendment was completed and granted Trust approval on 16^th^ November 2022.

An important component of this trial is for the research team to work closely with the clinical service, specifically the CPAP practitioners (a representative will be included in the TSC). It will provide a direct line of communication to the staff conducting recruitment to get real-time feedback from patients approached (and should hopefully aid with participant retention). The CPAP team’s understanding of the patient population at Papworth will also be extremely important when it comes to adapting the logistics of the recruitment process in the event of low recruitment; and for the future rollout of any changes to clinical practice that the trial results lead to.

## Assignment of interventions: allocation

### Sequence generation {16a}

Randomisation company Sealed Envelope will generate the allocation sequence. Permuted block randomisation will be used to randomise patients with random permuted block sizes. The allocation ratio of the combination therapy to CPAP is 1:1. There will be no stratification.

### Concealment mechanism {16b}

Any investigator seeking to randomise a patient to the trial has to answer screening questions and confirm consent on the online website. Once this is complete the system will release the randomisation allocation. Sealed Envelope provides a system and generates sequences independent of the trial team.

### Implementation {16c}

The randomisation service will be hosted by Sealed Envelope, who will generate the allocation sequence.

## Assignment of interventions: blinding

### Who will be blinded {17a}

Due to the nature of the intervention, patients and clinical staff cannot be blinded whilst the patient is receiving randomised therapy. However, a team of research staff will collect data on outcomes and these staff will be blinded. The interim analysis and sample size re-estimation will be done by an independent unblinded statistician so that the trial statisticians can remain blinded.

### Procedure for unblinding if needed {17b}

As above: due to the nature of the intervention, patients and clinical staff cannot be blinded whilst the patient is receiving randomised therapy. There is no need to unblind the trial statisticians until the trial analysis is complete.

## Data collection and management

### Plans for assessment and collection of outcomes {18a}

Papworth Trials Unit Collaboration (PTUC) Data Management team will provide data management oversight for the study and will coordinate with the Statistical and Health Economics teams to design the trial case CRF and to ensure data quality.

All data will be collated into the bespoke trial database within OpenClinica, including the sleep diary for the combination treatment arm. Participants will be given the option to access a patient portal (Participate) within OpenClinica to complete the daily sleep diary and questionnaires for follow-up visits conducted remotely. If paper sleep diaries are used the relevant data will be transcribed into the trial data base.

Treatment preference will be recorded in OC.

### Plans to promote participant retention and complete follow-up {18b}

As recruitment: the research team will work closely with the clinical service, specifically the CPAP practitioners (a representative will be included in the TSC). It will provide a direct line of communication to the staff conducting recruitment to get real-time feedback from patients approached, which we hope will aid participant retention. The availability of telephone support aims to deal with any concerns that could impact on participant retention in a timely fashion. We have anticipated there might be reticence to attend appointments in person at Papworth and therefore retained the option to conduct the research visits 2 and 3 remotely. To improve data collection for follow-ups, we have put in place processes for participants to record data on either paper or by using the personal electronic CRF system available to the study so they can complete the study questionnaires at a time that suits them, rather than during core working hours. In addition, there is an option for participants to send electronic images of the sleep diary entries to Papworth.

### Data management {19}

The investigator/clinical research team must maintain source documents (patient’s medical record) for each patient in the study, consisting of all demographic and medical information. A copy of the consent form and patient information sheet will also be uploaded into the patient’s medical record. All information in the CRFs, apart from the questionnaires, must be traceable to and consistent with the source documents in the patient’s hospital medical notes (ICH/GCP 4.9.2).

Full CPAP data downloads will occur at the end of each treatment arm. Bespoke reports of specific datasets will be generated as required and then entered directly into the eCRF.

On its return to Papworth, data from the WatchPAT device will be transferred to a secure server by research staff. The sleep study reports will be downloaded from this server as required and then directly entered into the eCRF.

### Confidentiality {27}

All Investigators and research staff must comply with the requirements of the Data Protection Act 2018 with regards to the collection, storage, processing, and disclosure of personal information and will uphold the Act’s core principles.

All data used in the formulation of trial reports will only contain anonymised data. The Data Management lead will ensure confidentiality of data is preserved when the data is transmitted to the Sponsor and Co-Investigators.

Patient identifiable information will be stored for a maximum of 12 months after the end of the study: the data will remain stored for 15 years as per the Trust policy. The study data will be exported from OpenClinica and archived locally on Royal Papworth Hospital NHS Foundation Trust servers.

### Plans for collection, laboratory evaluation and storage of biological specimens for genetic or molecular analysis in this trial/future use {33}

n/a: There will be no collection of biological specimens.

## Statistical methods

### Statistical methods for primary and secondary outcomes {20a}

#### Primary

The primary outcome is the difference in CPAP adherence (hours per night) between treatment arms. The adherence in combination therapy minus that in CPAP defines the difference.

The analysis will be a mixed-effects model, with a random intercept for each participant to account for variation in adherence within participants. We will adjust for oxygen desaturation, ESS at baseline, age, gender and BMI. If it is not possible to obtain adherence per night and instead we can only obtain mean adherence for each participant per treatment, the above analysis will not be possible. Instead, we will use a linear regression, adjusting for the same covariates as specified immediately above. In either case, we will report the 95% confidence interval for the difference in adherence between the two arms. If the lower bound of the 95% confidence interval is greater than 1 (hour), we will conclude that there is evidence of increased CPAP adherence on the experimental (CPAP + MAD) arm compared to the standard (CPAP) arm.

We will test for a period effect and for a treatment-by-period interaction using two-sample t-tests.

The statistical analysis will be reported according to CONSORT extension guidelines for adaptive trials.

In cases of missing data, the missing data mechanism will be explored, and multiple imputation may be applied as a sensitivity analysis as appropriate. Missing data for a night may indicate zero adherence for that night.

#### Secondary

Two-sample or paired t-tests will be used to analyse the following secondary outcomes:

- Mean CPAP pressure
- 4% ODI
- AHI
- Blood pressure
- Number of telephone support calls
- Time spent preparing and supporting/refitting MAD/CPAP
- Number of visits to secondary care
- Use of either primary or community care
- ESS (difference between baseline and each period end)
- FOSQ
- EQ5D (difference between baseline and each period end)
- SF-36 (difference between baseline and each period end)
- PSQI (difference between baseline and each period end)
- Patient satisfaction
- Treatment preference

Side effects will be summarised by tables per arm.

The statistical and health economic analysis plans will be included as updates to the protocol.

### Methods for economic analysis

The economic analysis will account for short-term cost-effectiveness, using a within-trial analysis, and long-term cost-effectiveness using a decision model. The within-trial cost-effectiveness and cost-utility analyses will be conducted from the viewpoint of the NHS, personal social services, and patients. Patient-specific resource use (device cost, device fitting and maintenance, delivery of kits and devices, use of GP and hospital services, treatment for adverse events) will be measured using data extracted from patient records and patient-reported data. Shared resource use (training of nurses and patients to use devices) will be accounted for using a mix of administrative logs and staff interviews. Valuation of resources used will be based on national unit costs or, in their absence, literature or local unit cost data. Outcome measures for quality of life (EQ-5D-5L, SF36) will be patient-reported and use NICE-recommended valuation methods, whereas effectiveness (adherence) will be device reported with no further valuation. A mixed effects model will be used to estimate differences in patient costs and outcomes (i.e. adherence, QALYs). We will investigate the importance of controlling for the following variables at baseline; quality of life, health service use, time period, oxygen desaturation, ESS at baseline, age, gender and BMI. We will undertake deterministic sensitivity analysis to consider, for example, varying the price and length of life of the MAD, and the impact of missing value imputation on findings. Results will be reported as total costs and effects for each arm, incremental cost-effectiveness ratios, cost-effectiveness acceptability curves and incremental net benefit.

The structure of the long-term cost-effectiveness model will be based on McDaid et al. [14], and account for the long-term impacts, e.g. on hypertension, stroke, and road traffic accidents. The model will be parameterised using; data from the PAPMAT trial (on treatment effects, costs, health utilities, the relationship between ESS and quality of life), updated national data (for mortality), estimates from McDaid et al. where a previous update did not find better data despite reviews of hundreds of abstracts (i.e. CVD risk, OSAS, road traffic accident risk), and a new evidence review on compliance. Deterministic sensitivity analysis will include; alternative estimates from the McDaid et al. model and Sharples et al. 2014 HTA report, alternative appropriately inflated costs from Sharples 2014 report. Probabilistic sensitivity analysis will be undertaken to assess uncertainty that will be illustrated using cost-effectiveness acceptability curves.

### Interim analyses {21b}

The proposed interim analysis will be conducted when 29 patients have completed the primary endpoint, which is expected to happen at Month 21. The primary rationale behind the timing of the interim analysis is based on the minimum sample size needed to make precise estimates of the variance parameters in each arm of the trial. A range of published literature exists on the topic of minimum sample size for pilot studies, which is akin to the sample size requirements for an interim sample size re-estimation or internal pilot.

Traditionally a “rule of thumb” approach was used to set the sample size for pilot studies at around 30, though there have also been a number of papers published giving a more scientific basis for the selection of sample size for pilot studies.

For two-arm studies with a continuous outcome, Julious [19] recommends a total sample size of 24 and Keiser [20] recommends a total sample size of 20–40, whilst Sim [21] recommends a total sample size of at least 50 and Teare [22] recommends a total sample size of at least 70. Our recommendation for performing the interim sample size re-estimation after 29 patients have completed their primary endpoint sits towards the lower end of these figures. This must be carefully balanced with the duration of treatment and follow-up, particularly in the case of trials using a cross-over design, which will naturally delay the point at which the interim analysis occurs.

Sample size re-estimation is the primary task of the interim analysis. The trial statistician will prepare the analysis code to calculate the standard deviations (SD) of the crossover difference in the primary endpoint between the CPAP/MAD combination therapy and the CPAP therapy where the treatment group is labelled using a dummy randomisation list. An independent statistician will then run the code with the real randomisation list provided by the data manager and output the updated estimates of the SD for the crossover difference. The original sample size calculation will be repeated with the updated estimates to provide an updated sample size estimate which will be reported to the DMEC.

Another important task is to present a summary of the safety data so that the DEMC can evaluate if there is evidence of treatment safety or treatment harm. The frequencies of the AEs and SAEs will be summarised by the treatment group. Note that AEs that are OSA symptoms will not be reported.

In addition, information about patient recruitment and non-compliance will be reported at the interim analysis. The summary of recruitment data will be presented as well as the treatment group and month where appropriate. Non-compliance will be summarised by treatment group and the reasons for non-compliance will be reported where appropriate.

The trial statisticians and data management team will assess data quality to ensure collecting high-quality data. The completeness of study data and data completeness will be monitored regularly.

Treatment efficacy will not be assessed at the interim analysis. In the event that the DMEC request additional data analyses at the interim stage the trial statistician will be responsible for providing these (via the independent statistician if unblinding is required).

### Methods for additional analyses (e.g. subgroup analyses) {20b}

The primary analysis is an adjusted analysis, intention to treat. The only additional analysis planned beyond the primary and secondary analyses is a subgroup analysis for ODI categories, comparing treatment compliance.

### Methods in analysis to handle protocol non-adherence and any statistical methods to handle missing data {20c}

Missing data will be quantified per variable (%). All essential variables for the outcomes are expected to be complete or at least low missing percentage before starting analysis.

For participants who discontinue the allocated treatment CPAP + MAD but continue to use the CPAP machine, we will use their adherence data as if they were still continuing their allocated treatment (i.e. intention to treat). For participants who discontinue the allocated treatment CPAP (and thus no longer provide adherence data), we will impute their adherence as zero hours for each remaining day. This pragmatic approach reflects the primary endpoint, which is the number of hours of CPAP adherence, and the wider research question, which is to examine if CPAP adherence is greater for participants allocated to CPAP + MAD compared to those allocated to CPAP only. More details with regards to the treatment of missing data will be given in the SAP, and in the health economics analysis plan (HEAP) in terms of costs.

### Plans to give access to the full protocol, participant-level data and statistical code {31c}

The protocol and statistical analysis plan (SAP) will be published open access, before the completion of the trial. The statistical code used will be available online (through the software website GitHub) within 12 months of publication of results.

## Oversight and monitoring

### Composition of the coordinating centre and trial steering committee {5d}

#### Co-ordinating centre

The trial is managed by Papworth’s Trials Unit Collaboration (PTUC), which is a collaboration with the MRC Biostatistics Unit, Cambridge, and the Department of Population Health Sciences, King’s College London. The Unit is fully registered with the UK Clinical Research Collaboration (No: 60). PTUC contributed to the overall study design, statistical and health economic design. It will oversee the study and provide project management oversight, data management, statistical and health economic analysis and research governance support.

#### Trial Steering Committee

A Trial Steering Committee (TSC) will be led by an Independent Chair. As per NIHR guidelines, the TSC will be composed of statistician, health economist, data manager, clinician, plus a Papworth patient representative and non-Papworth representation, including a Sponsor representative and a member of the CPAP Practitioner team. The interim analysis will be carried out by a statistician who is independent of the study.

The TSC will meet at 6-monthly intervals to monitor and supervise the trial, to ensure it is being conducted according to the protocol and timelines, to review any relevant information from other sources (e.g. other related trials) and to consider recommendations made by the Data Monitoring and Ethics Committee (DMEC).

### Composition of the data monitoring committee, its role and reporting structure {21a}

The DMEC will be led by an Independent Chair who is an expert in the field. As per NIHR guidelines, the DMEC will include an independent expert Statistician and a Clinician.

Annual DMEC meetings will review progress against the agreed milestones, recruitment and safety. The independent DMEC will: (1) review the assumptions underlying the sample size calculations and determine whether additional interim analyses of trial data should be undertaken; (2) develop clear, robust safety stopping rules based on regular (at least yearly) adverse event monitoring; (3) consider results of other interim analyses and relevant information arising elsewhere; (4) consider any requests for the release of interim trial data and advise the trial steering committee on this; and (5) make recommendations to the trial steering committee about continuation of recruitment. The trial statistician will provide the interim reports for the DMEC.

### Adverse event reporting and harms {22}

#### Adverse events

All AEs will be recorded in the patient’s medical records.

Expected harms include but are not limited to:

- Broken tooth/crowns
- Alteration of bite
- Bleeding gums

Both expected and unexpected harms will be assessed non-systematically. Participants will be asked to record any AEs they experience in a diary during the combination CPAP-MAD arm. They will also be asked about AEs in open-ended questions at the end of each treatment arm (visits 4 and 5). All expected and unexpected harms considered to be of clinical significance in terms of direct health-related consequences or indirectly through impacting adherence to treatment will be reported.

AEs that are OSA symptoms will not be reported.

#### Definition of serious adverse events

A serious adverse event is any untoward medical occurrence that:

- Results in death
- Is life-threatening
- Requires inpatient hospitalisation or prolongation of existing hospitalisation
- Results in persistent or significant disability/incapacity

The investigators do not believe that this study places participants at risk of a serious adverse event.

#### Reporting procedures for serious adverse events

Any serious adverse event (SAE) occurring to a participant will be reported to the REC that gave a favourable opinion of the study where in the opinion of the Chief Investigator the event was “related” (resulted from administration of any of the research procedures) and “unexpected” in relation to those procedures.

Reports of related and unexpected SAEs will be submitted within 15 working days of the Chief Investigator becoming aware of the event and using the appropriate documentation.

### Frequency and plans for auditing trial conduct {23}

A monitoring plan for the trial is designed prior to the trial opening and is agreed by the TSC. The initial monitoring takes place soon after recruitment begins and then at six monthly intervals (unless there is a reason to increase the frequency). The trial is audited to determine whether trial-related activities are in accordance with protocol, SOPs and GCP as part of the R&D Department’s ongoing program of audits. The monitoring and audits are undertaken by appropriately trained staff from the Royal Papworth R&D department, independent of the Trials Unit and trial investigators, Monitoring and audit findings are reported to the R&D Quality & Audit Committee, which in turn reports to the R&D Directorate Committee.

### Plans for communicating important protocol amendments to relevant parties (e.g. trial participants, ethical committees) {25}

Before the start of the study, or implementation of any amendment, approval of the trial protocol, protocol amendments, informed consent forms and other relevant documents will be obtained from the Regional Ethics Committee (REC) and Health Research Authority (HRA). All correspondence with the REC and RfPB will be retained in the Trial Master File (Sponsor File/Investigator Site File).

### Dissemination plans {31a}

We will make the findings of the study publicly available through publication or other dissemination tools without any unnecessary delay and an honest accurate and transparent account of the study will be given. Any discrepancies from the study as planned in this protocol will be explained.

## Discussion

Recruitment will be consecutive from the centre’s regular clinical population. Most patients are referred from primary care (although from a single centre) and so this, in addition to the fact that our approach to treating OSA is not significantly different to that of other centres, give us confidence that the results of this trial will be generalizable.

The inclusion criteria include a pragmatic approach to classifying OSA severity, depending on the diagnostic test used. Overnight oximetry is our centre’s entry level diagnostic test. It is used alongside expert clinical assessment to confirm OSA in most of our patients who receive this diagnosis and go on to start CPAP. Oximetry provides a 4%ODI/h. This is less sensitive than respiratory polygraphy or polysomnography, both of which also provide an AHI/h. Even using the AHI there are differing sensitivities etc. depending on the scoring rules used. Despite this, it is common practice to use the same numerical categories to define OSA severity. The impacts of this variability on diagnostic sensitivity etc. are well recognised. Whilst this might be relevant in other studies of OSA interventions using different outcomes, we do not consider it to be a problem for this study, where the primary outcome is adherence to treatment. Severity categories based on sleep study indices (4%ODI or AHI) are arbitrary and correlate poorly with symptom severity and adherence. Taking only patients with at least moderately severe OSA means there is negligible risk of including subjects with a false positive diagnosis, particularly as those who have not responded to initial auto-titrating CPAP will be identified and their diagnosis reviewed at visit 2 as part of eligibility checking.

We hope that most participants will be willing to attend all visits in person. This will allow, among other benefits, the monitoring of blood pressure, which is an important secondary outcome that continues to be the subject of ongoing meta-analyses. However, to be inclusive and maximise recruitment and retention we will be offering visits 4 and 5 remotely to those patients who might otherwise find it difficult to commit to the trial. This is likely to also increase generalizability as data from our centre suggest that patients from areas of higher social deprivation may be less likely to attend appointments and remain under clinical follow-up.

## Trial status

Protocol V5.0, 5^th^ April 2023.

Recruitment began: October 2022.

Recruitment will complete: January 2025 (approximately).

## Data Availability

The Principal Investigator will have access to the final dataset. The trial statisticians will have access to a blinded full dataset for analysis, whilst the health economists will have access to an unblinded full dataset for analysis. Upon request, we will provide access to a pseudo-anonymised final dataset.

## Abbreviations

AHI: Apnoea-hypopnoea index
CPAP: Continuous positive airway pressure
CRF: Case Report Form
CM: Conservative Management
CTC: Clinical Trial Coordinator
DMEC: Data Monitoring and Ethics Committee
ESS: Epworth Sleepiness Score
EQ-5D: EuroQoL-5D
FOSQ: Functional Outcomes of Sleep Questionnaire
GCP: Good Clinical Practice
HRA: Health Research Authority
MAD: Mandibular Advancement Device
MCID: Minimal Clinical Important Difference
MSLT: Multiple Sleep Latency Test
NIHR: National Institute of Health Research
NRES: National Research Ethics Service
ODI: Oxygen Desaturation Index
OSA: Obstructive sleep apnoea
OSAS: Obstructive sleep apnoea syndrome
PIL: Participant/Patient Information Leaflet
PPI: Patient and Public Involvement
PRA: Patient Research Ambassador
PSQI: Pittsburgh Sleep Quality Index
PTUC: Papworth Trials Unit Collaboration
QALYs: Quality adjusted life years
QoL: Quality of life
REC: Research Ethics Committee
RSSC: Respiratory Support and Sleep Centre
SATA: Sleep Apnoea Trust Association
SF-36: Short Form-36
SOP: Standard Operating Procedure
TSC: Trial Steering Committee
